# A cluster randomised feasibility trial evaluating six-month nutritional interventions in the treatment of malnutrition in care home-dwelling adults: recruitment, data collection and protocol

**DOI:** 10.1186/2055-5784-1-3

**Published:** 2015-01-12

**Authors:** Ruth Stow, Alison Rushton, Natalie Ives, Christina Smith, Caroline Rick

**Affiliations:** 1 The Heart of England NHS Foundation Trust Nutrition Support Service, 3 The Green, Solihull, B90 4LA UK; 2 University of Birmingham, School of Sport, Exercise and Rehabilitation Sciences, Edgbaston, Birmingham, B15 2TT UK; 3 University of Birmingham, Birmingham Clinical Trials Unit, College of Medical and Dental Sciences, Public Health Building, University of Birmingham, Birmingham, B15 2TT UK; 4 Language and Communication, Division of Psychology and Language Sciences, University College London (UCL), 202d Chandler House, 2 Wakefield Street, London, WC1N 1PF UK

**Keywords:** Malnutrition, Nutrition support, Oral nutritional supplements, Sip feeds, Care homes, Malnutrition Universal Screening Tool (MUST), Elderly, Nutritional intervention, Nutritional risk

## Abstract

**Background:**

Protein energy malnutrition predisposes individuals to disease, delays recovery from illness and reduces quality of life. Care home residents are especially vulnerable, with an estimated 30%–42% at risk. There is no internationally agreed protocol for the nutritional treatment of malnutrition in the care home setting. Widely used techniques include food-based intervention and/or the use of prescribed oral nutritional supplements, but a trial comparing the efficacy of interventions is necessary. In order to define outcomes and optimise the design for an adequately powered, low risk of bias cluster randomised controlled trial, a feasibility trial with 6-month intervention is being run, to assess protocol procedures, recruitment and retention rates, consent processes and resident and staff acceptability.

**Methods:**

Trial recruitment began in September 2013 and concluded in December 2013. Six privately run care homes in Solihull, England, were selected to establish feasibility within different care home types. Residents with or at risk of malnutrition with no existing dietetic intervention in place were considered for receipt of the allocated intervention. Randomisation took place at the care home level, using a computer-generated random number list to allocate each home to either a dietetic intervention arm (food-based or prescribed supplements) or the standard care arm, continued for 6 months. Dietetic intervention aimed to increase daily calorie intake by 600 kcal and protein by 20–25 g.

**Results:**

The primary outcomes will be trial feasibility and acceptability of trial design and allocated interventions. A range of outcome assessments and data collection tools will be evaluated for feasibility, including change in nutrient intake, anthropometric parameters and patient-centric measures, such as quality of life and self-perceived appetite.

**Conclusions:**

The complexities inherent in care home research has resulted in the under representation of this population in research trials. The results of this feasibility trial will be used to inform the development and design of a future cluster randomised controlled trial to compare food-based intervention with prescribed oral nutritional supplements (ONS) in the treatment of malnutrition within the care home population.

**Trial registration:**

Current Controlled Trials ISRCTN38047922

**Electronic supplementary material:**

The online version of this article (doi:10.1186/2055-5784-1-3) contains supplementary material, which is available to authorized users.

## Background

Often under recognised and under treated, protein energy malnutrition (PEM) develops when energy intake and/or protein intake chronically fail to meet the body’s nutritional requirements [1]. Commonly described as both a cause and a consequence of adverse outcomes, PEM predisposes individuals to disease and delays in recovery from illness [2]. In UK, more than three million people are either malnourished or at risk of malnutrition and in 2007, the associated health and social care costs were estimated to exceed £13 billion annually, more than 10% of the public expenditure on health care [3].

Care homes, which provide residential accommodation, together with nursing or personal care are arguably home to one of our most vulnerable populations [4], 30%–42% of whom are believed to be at risk of PEM [5] and most of whom have multiple physical and mental health and social care needs. PEM has significant negative impacts on the physical and emotional well-being of care home residents and has been linked to increased vulnerability to infection and pressure ulcers, clinical complications, depression, anxiety and a decreased quality of life [6, 7].

Evidence for nutritional strategies to address malnutrition in the care home setting is lacking [8]. Widely used dietetic techniques to enhance oral dietary intakes include food-based intervention (recipe enrichment or fortification to increase energy and/or protein density, flavour enhancement, provision of nourishing snacks and/or fortified drinks) and/or the use of prescribed oral nutritional supplements (ONS) [9], considered to be ‘dietary foods for special medical purposes’ (FSMPs) [10]. The British Dietetic Association (BDA) and The National Prescribing Centre (NPC) advocate improving nutritional intake first using enrichment of conventional food and secondly by prescribed means [11, 12]. However, few trials have evaluated the food-based approach and it remains unclear whether this intervention is able to improve clinical and functional outcomes for malnourished individuals [8, 13, 14].

The use of nutrition support interventions for the treatment of malnutrition has received growing attention within the literature, with the majority of studies using prescribed ONS as the main nutritional intervention strategy [15–19]. Existing systematic reviews of malnutrition interventions [9, 20–23] have tended to focus on the effectiveness of ONS intervention compared with placebo or usual care, predominantly within the acute setting. Reviews of the efficacy of ONS in the treatment of malnutrition have demonstrated improved nutritional status when compared to usual care, but the current level of evidence suggests that the length of intervention of the reviewed studies is often too short to be able to detect differences in morbidity, functional status and quality of life.

A Cochrane review and meta-analysis conducted in 2007 and updated in 2011 aimed to address the impact of food-based intervention and dietary advice on PEM, compared with usual care or ONS [8]. The evidence from the review suggests that dietary advice and intervention with or without ONS for the treatment of malnutrition may improve weight and indicators of muscle mass. However, whilst most included trials provided information on the duration of the intervention, there was almost no information on the nature, intensity and content of the food-based interventions. The review also highlighted a complete lack of evidence for the effects of food-based intervention on patient-reported outcome measures (PROMs) such as quality of life (QoL), which may be an important determinant of intervention effectiveness.

No systematic review to date has made any specific conclusions regarding nutritional interventions for the treatment of malnutrition in the elderly care home setting. There is a tendency to avoid research in care homes, because of the challenges and methodological issues involved in conducting research in this setting [24]. Many research studies specifically exclude care home residents on the basis that their inclusion would present the research team with ethical and practical difficulties [25]. Studies, which have been conducted in the care home setting often, include dementia and immobility within the exclusion criteria [26–28], despite these being well-established risk factors for malnutrition. As a result, knowledge on the actual effectiveness of nutritional intervention in this vulnerable population is limited and the clinical applicability of findings to those care home residents most at risk of malnutrition is often questionable. With an ageing population set to increase in the coming years, care homes will play an increasingly vital role in supporting and caring for older people. The lack of evidence to support the best practice has led to recommendations for more studies to be conducted in the care home setting [29] and provides an opportunity to bring new ideas to the field.

To enable the efficacy of food-based interventions to be compared with ONS intervention within a care home population, an adequately powered RCT is required, using interventions that are equivalent in energy and protein composition, alongside a comparative routine care arm. The Medical Research Council (MRC) framework [30] explicitly recommends that complex interventions should be investigated using preliminary studies, prior to evaluation in a RCT, in order to optimise the trial design, to define the outcomes and to ensure feasibility. Given the expected complexities of care home research, a feasibility trial for a multi-centre cluster RCT was proposed, to explore trial design, staff, and resident acceptability and to provide data to estimate the parameters required to design a definitive RCT.

## Methods/design

This protocol has been reported in reference to the CONSORT guidelines [31].

### Research ethics approval

To meet the requirements of the Research Governance framework, approval for the trial was required from the Research Ethics Committee (REC) and the Research and Development (R&D) Department of the NHS Trust for sponsoring the research. This protocol was submitted to the West Midlands NHS Local Research Ethics Committee for proportionate ethical review and obtained approval prior to commencement (see Additional file 1).

The REC requested for randomisation and intervention allocation to take place at the level of the care home. The committee determined that participation in the collection of participant-reported outcome measures (PROM’s) within the feasibility trial would not be of benefit to the wider population of adults that lack capacity, and their inclusion in PROMs could not be justified in accordance with the Mental Capacity Act [32].

The protocol was also submitted to the Research and Development Department of the Heart of England NHS Foundation Trust for Research governance approval. This was received on 17 October 2013 (see Additional file 2).

### Aims and objectives

The primary aim is to assess the feasibility of conducting a definitive trial in UK care home setting, in terms of recruitment and retention, use and acceptability of the nutritional interventions, follow-up at 3 and 6 months and the feasibility and acceptability of outcome measures and data collection methods.

The following criteria will be used to assess success:

1. Recruitment target for care homes (six) and for residents (*n* ≥ 50) met in the time available (3 months)
2. Favourable difference shown in the number of residents at risk of malnutrition and the number deemed eligible to participate (≤20%)
3. Retention rate at 100% for care homes, at 6-months follow-up and at 65% or more for residents, accounting for the expected high mortality and attrition rate
4. Intervention crossover of less than 10% for each intervention
5. More than 80% of residents to be compliant with 50% of the dietetic-led intervention dose (≥300–450 kcal) and more than 60% of residents to be compliant with 75% or more of the dietetic-led intervention dose (≥450–600 kcal)
6. 85%–90% staff adherence to intervention schedule
7. Data completeness of ≥80% for each outcome measure and data collection method

Components of the trial that are deemed to be infeasible or unacceptable will be modified in the definitive trial or will be removed altogether.

The trial will also examine, qualitatively, the acceptability of the interventions, outcome measures and data collection methods to malnourished care home residents and care home staff.

The trial will have a number of quantitative objectives:

1. To assess how many care homes for the elderly accept the invitation to participate in a nutritional intervention feasibility trial
2. To determine whether the eligibility criteria for malnourished care home residents are too open or too restrictive, by estimating feasible eligibility and recruitment rate
3. To assess retention of care homes and residents, by estimating 3- and 6-month follow-up rates
4. To investigate the acceptability of nutrition support interventions to malnourished care home residents, in terms of compliance, and to care home staff in terms of adherence to the intervention schedule
5. To determine the acceptability and feasibility (and factors influencing this) of the different outcome measures as methods to measure efficacy of nutritional interventions within a definitive trial
6. To investigate the completion and accuracy of nutritional screening and questionnaire completion by care home staff
7. To determine how many malnourished care home residents are able to participate in PROMs and to complete the questionnaires
8. To measure key outcome domains (for completion rates, missing data, estimates, variances and 95% confidence intervals for the difference between the intervention arms) for malnourished care home residents including physical outcome measures and PROMs
9. To collect and synthesise data, from which to estimate the intracluster correlation coefficient (ICC) and inform the sample size of a definitive trial

## Trial status

A prospective cluster randomised feasibility trial with 6-month intervention. A sequential, explanatory mixed method design was proposed, beginning with a quantitative method to assess trial feasibility (phase 1), followed by a qualitative method, involving detailed exploration with a few individuals [33] (phase 2).

A cluster trial design has been chosen primarily to avoid contamination [34], because the assigned care staff receiving additional training and support cannot be expected to treat individual residents differently, but it is also being used for practical reasons. The aim of the trial is to assess the feasibility and acceptability of delivering and evaluating nutritional interventions for malnutrition in the care home setting, and hence the home is the unit of randomisation.

### Phase 1

Recruitment for phase 1 of the trial began in September 2013 and concluded in December 2013. The quantitative phase follows a controlled experimental design, collecting data on changes in anthropometric markers and nutrient intake along with the use of quantitative questionnaires. The trial needed to be open label due to the nature of the nutritional interventions under investigation.

### Phase 2

Recruitment for phase 1 of the trial began in July 2014 and is expected to conclude in October 2014. Following phase 1, the quantitative findings will be further explored with a sample of residents and care home staff, using semistructured interview and focus group techniques. The qualitative findings will be used to complement, explore and explain the quantitative data collection and will ensure that resident and staff perspectives are used to inform the future trial design.

### Setting and population

The feasibility trial is being conducted within the borough of Solihull, West Midlands, in England. Prior to trial commencement, 17 care homes in Solihull, providing accommodation for older people, were receiving regular dietetic input, to improve the identification and first-line nutritional management of malnutrition. Six privately owned care homes were invited to take part in the trial. The care homes were purposively sampled to obtain a diverse sample based on ownership, size and type of care provided (nursing and/or residential), to enable the evaluation of feasibility and acceptability of the trial design and methodologies across a range of long-term care settings.

### Participants

#### Phase 1

**Figure 1:**
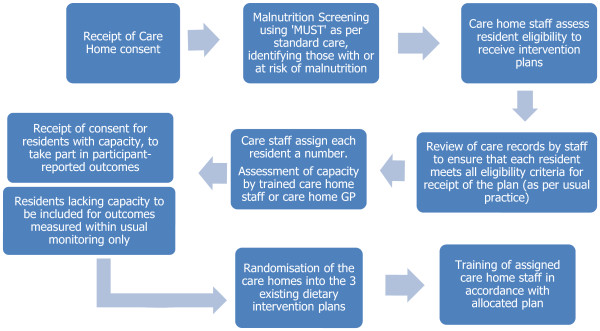
**The recruitment and consent process.**

Eligible residents within each of the participating care homes were identified using routine malnutrition screening, a review of care home medical records and consultation with care staff (see Figure 1). Two assigned care home staff (nurses or senior carers) within each care home site conducted nutritional screening using the Malnutrition Universal Screening Tool (MUST) [35] to identify those with or at risk of malnutrition. ‘MUST’ classifies risk as low, medium or high on consideration of current body mass index (BMI), history of unintentional weight loss (%) and acute illness effect. Monthly screening of all residents by nursing or senior care staff was already a standard practice within the selected care homes. ‘MUST’ has been validated for use in adults, has very good to excellent inter-observer reliability in care homes (kappa values of 0.8–1.0) and has been found to be acceptable to both participants and healthcare workers [35].

**Figure 2:**
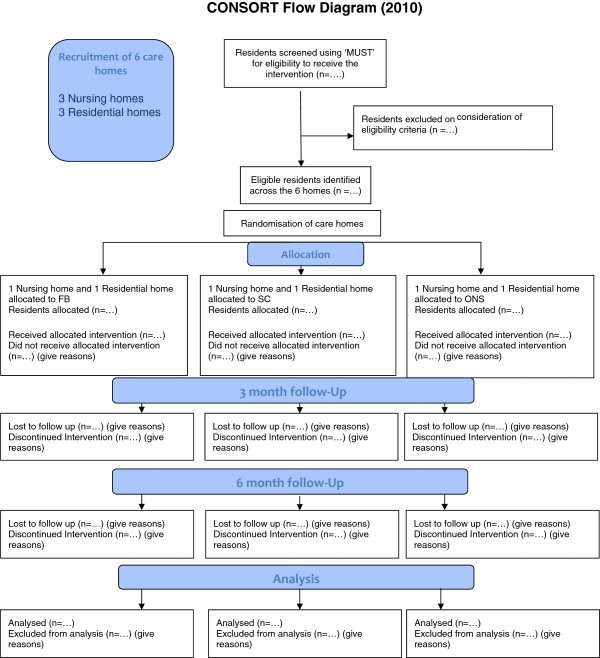
**The expected flow of participating residents through the feasibility trial.**

Whilst some areas may advocate dietetic assessment for patients scoring high risk, it is now more common for dietetic services to recommend that care homes implement first-line advice and possibly a trial with sip feeds prior to considering dietetic referral. This trial has been designed to reflect usual practice within these care homes, therefore dietetic assessment was not conducted following identification using ‘MUST’. The care home staff were provided with a screening log, with a checklist of the inclusion and exclusion criteria (below). The staff reviewed the care records of those residents identified as medium or high risk of malnutrition, against the eligibility criteria, consulting with the Solihull Nutrition support dietetic service and the care home GP, as required. Each eligible resident was assigned a unique trial number, and the GP for each participating care home site was informed in writing of the involvement of the care home in the study (with the care home consent to do so) (see Figure 2).

Inclusion criteria for residents:

1. A score of ‘1’ or higher on the Malnutrition Universal Screening Tool (MUST)
2. Able to eat and drink
3. Registered with a Solihull GP and subsequently eligible for the provision of healthcare services provided by the Heart of England NHS foundation Trust (HEFT)
4. Aged over 65 years

Exclusion criteria for residents:

1. Receiving (or likely to receive in the next 6 months) enteral tube feeding or parenteral nutrition
2. Receiving nutrition support in the form of individualised dietetic advice or prescribed ONS
3. Have a known eating disorder or illness, which requires a therapeutic diet incompatible with fortification and/or supplementation. This may include but is not limited to galactosemia or diagnosed lactose intolerance, chronic renal disease requiring dialysis, poorly controlled diabetes, in receipt of active cancer treatment or liver failure
4. On an end-of-life care pathway

Exclusion criteria for participant-reported outcome measures (PROMs):

1. Non-native English speaking
2. Lacking the capacity to consent

It was anticipated that the population of residents identified with or at risk of malnutrition in each care home would include individuals lacking the capacity to consent [4]. In accordance with the requirements of the approving REC, residents that lacked capacity were excluded from taking part in PROMs. The decision to exclude non-native English speaking residents with capacity from participation in the collection of PROM’s was based on existing knowledge and experience of the population group and a consideration of the finances available to run the feasibility trial. The primary researcher works clinically as a dietitian within the Solihull borough and estimated that less than 5% of the resident and staff population are non-native English speaking. This feasibility trial is being conducted as a student Master’s project. Due to limited resources, interpreter services could not be engaged.

### Individual consent for PROM’s

Written consent was sought on an individual basis from those residents that have been assessed by trained care home staff or the care home GP as having capacity. Each resident was provided with a participant information sheet and was given 1 week to read, ask further questions and decide whether they would like to provide information on quality of life, health state and dietary satisfaction through questionnaires and self-reported scales. Each resident was then asked to sign a consent form.

As the screening and consent process took place prior to cluster randomisation, the residents, care home staff and the researcher were all blinded to the allocated intervention. This ensured that decisions regarding eligibility and consent were not influenced by the nutritional intervention allocated to the care home site.

#### Phase 2

Following data collection of PROMs for phase 1, those residents that were indicated on the consent form that they would like to be considered for individual interviews will form the sampling frame for potential inclusion within the qualitative phase. A non-random method of purposive sampling involving care home staff discussions and consideration of the PROMs data will be used to identify two to three potential participants per care home for individual semistructured interviews with the primary researcher.

A focus group of six to eight staff will be identified within each care home site that has management approval for staff participation. Care home staff will be selected on the basis that they have participated in the trial and will therefore have something to say. Separate information sheets and consent forms for residents and staff have been developed for the qualitative phase and will be presented to eligible residents and staff prior to their inclusion.

### Interventions

Assigned care home staff within each care home received dietitian-led training to support delivery of the 6-month allocated intervention and/or a refresher on current standard care.

#### Food-based intervention

The content of the food-based intervention was based on the developed resources and guidelines of the Solihull Nutrition Support Project, the PrescQIPP Nutrition Toolkit [36] and the Malnutrition pathway [37], which was developed and agreed by a multi-professional consensus panel. Assigned care staff within the care homes randomised to food-based intervention received face-to-face instruction by the primary researcher to increase participating resident’s daily nutritional intake by 600 kcal and 20–25 g protein, using agreed interventions, alongside the standard care home nutritional intervention for malnutrition. Food-based interventions included the provision of homemade enriched drinks between meals and/or the provision of between-meal fortified snacks. The intervention combination agreed by the primary researcher and the care home staff was documented for each resident, at baseline and at 3 months. The intervention recipes and a breakdown of the calorie and protein content were provided to the care home staff and catering teams.

The primary researcher continued to make dietetic visits to the care homes on a monthly basis and determined compliance with the intervention at 3 and 6 months. The care home staff were required to record resident intake of the food-based intervention on the daily food record chart, as a proportion taken. The average intake will be calculated from a three non-consecutive records at 3 and 6 months. Compliance will be categorised into ‘compliant’, if 75% or more of the advised food/beverage is consumed daily and ‘non-compliant’ if less than 75% is consumed.

#### ONS intervention

Assigned care home staff within the two care homes allocated to dietetic-led ONS intervention received instruction by the primary researcher to increase the daily nutritional intake of participating residents by approximately 600 kcal and 24 g protein, alongside the standard care home nutritional intervention for malnutrition. This nutrient increase was achieved through the consumption of two liquid ONS daily.

The Medicines and Healthcare products Regulatory Agency (MHRA) define ONS as Non Investigational Medicinal Products (NIMPs). The ‘standard’ ready to drink ONS is comprised of a combination of macronutrients and micronutrients and is presented in liquid form. These widely used supplements provide approximately 300 kcal and 12 g of protein per serving. Three ‘standard’ ONS were used within this trial: Fortisip 200-ml bottle, Fortisip Compact 125-ml bottle and Nutriplen 125-ml bottle. A bottle/serving of each supplement is equivalent in energy and protein content (Table 1). Fortisip bottle and Fortisip compact are manufactured by Nutricia Advanced Medical Nutrition and were provided by Nutricia for use within the first 3 months of the intervention duration. Nutriplen is manufactured by Nualtra Ltd and was provided by Nualtra for the second 3 months of the intervention duration. All of the ONS were provided to residents under the control of a registered dietitian.

**Table 1:** **Oral nutritional supplement composition**

ONS	Volume (ml)	Kcal/ml	Energy content (kcal) per serving	Protein content (g) per serving
Fortisip bottle (Nutricia)	200	1.5	300	12
Fortisip compact (Nutricia)	125	2.4	300	12
Nutriplen (Nualtra)	125	2.4	300	12

Nutritional information taken from http://www.nutricia.ie/products and http://nualtra.ie/information-for/dietitian.

The primary researcher continued to make dietetic visits to the care homes on a monthly basis and determined compliance with the intervention at 3 and 6 months. The care home staff were required to record intake of ONS on the daily drugs chart, as a proportion taken. The average intake will be calculated from three non-consecutive charts at 3 and 6 months. Compliance will be categorised into ‘compliant’, if 75% or more of the advised dose is consumed daily and ‘non-compliant’ if less than 75% is consumed.

#### Standard care arm

The purpose of the standard care home first-line intervention is to provide an energy-dense diet [38], through the provision of small, frequent meals and/or recipe enrichment with additional calories, alongside prompting and assistance from care home staff where required. Standard care was provided within all six care homes, to ensure that no resident at risk of malnutrition was denied access to first-line treatment. Eligible residents within the two care homes allocated to standard care only received an energy-enriched diet, in line with the training already provided to care home staff (including catering teams), by the local nutrition support dietetic service. The care homes continued to receive monthly dietetic visits from the primary researcher, but individualised advice or plans were not provided.

### Safety considerations

#### Risk of re-feeding syndrome on initiation of an energy-fortified diet or ONS

Those at risk of re-feeding syndrome were identified through routine ‘MUST’ screening, if they had any of the following: a BMI of less than 16 kg/m [2], weight loss of greater than 15% over the last 3 to 6 months, no or negligible dietary intake for ten consecutive days [9]. If any resident was identified as being at risk of re-feeding syndrome at baseline, nutritional intervention was commenced cautiously, at 10 kcal/kg/day, increasing to provide the additional 600 kcal and 20–25 g protein by day 7. The dietitian also requested that the GP monitors electrolytes and glucose. This procedure is in line with usual, standard dietetic practice and national guidance [9].

#### Deteriorating swallow function (dysphagia) and an increased risk of aspiration

If dysphagia was identified or suspected during the 6-month intervention duration, by the care home staff or the primary researcher, this prompted a referral to the Speech and Language Therapy (SaLT) team, as per usual standard practice.

#### Decline of nutritional status in the standard care arm

If any resident in receipt of standard care without added intervention suffered a significant decline of nutritional status, dietetic intervention (food-based or ONS) was considered after 6 weeks of standard care, as per local and national best practice guidelines.

### Outcome measures

#### Primary outcome measures

Feasibility considerations:

- Recruitment of care homes (ratio between those who consented to participate and those who were approached)
- The suitability of resident eligibility criteria (ratio between those who were considered eligible to enter the trial and those who were at risk of malnutrition)
- Residents’ willingness to participate in PROM’s (ratio between those who consented to participate and those who were eligible and approached)
- Resident retention in the trial (ratio between those who entered the trial and those that remained at 3 months and at 6 months)
- Data collection (ratio between completed questionnaires/screening tools/record charts and non-completed or unavailable questionnaires/screening tools/record charts) and physical outcome measurements (ratio between those residents measured and those that could not be measured)

Acceptability considerations:

- Resident acceptability of allocated interventions (as measured by compliance and crossover rate in each intervention arm)
- Staff acceptability of allocated interventions (as measured by adherence to intervention plan)
- Resident acceptability of physical measurements (as measured by the ratio between those residents measured and those that refused)
- Resident acceptability of PROM’s data collection tools: appetite and dietary satisfaction VAS tool, EQ5D VAS and questionnaire, COOP quality of life tool. Questionnaire completion rates will be calculated

Secondary outcome measures:

- Changes in nutritional status (malnutrition risk score, weight, body mass index, mid arm muscle circumference), functional status (hand grip strength), nutritional intake (energy, protein, fluid), healthcare resource usage and PROM’s
- Usefulness of resident completed questionnaires and tools (appetite and dietary satisfaction VAS, EQ5D VAS and questionnaire, COOP tool) and staff completed questionnaires and tools (sMMSE, healthcare resource usage). The ratio between those residents in the trial with capacity and those residents in the trial, but lacking capacity will be recorded. Questionnaire completion rates will be calculated and any third-party help used in completion will be recorded

### Descriptive variables

Following confirmation of resident eligibility, the staff in each care home completed a baseline assessment form for each resident. This form included the following information:

1. Resident Trial Number (assigned by care home staff following screening)
2. Resident gender
3. Care home type (nursing, residential)
4. Primary diagnosis
5. Diagnosis of dysphagia (Yes or No). If Yes, to indicate recommended food and fluid modifications
6. Diagnosis of dementia (Yes or No)
7. Risk of re-feeding syndrome (Yes or No). If yes, to indicate risk factor(s)
8. Capacity (Yes or No)
9. Informed consent received (Yes or NO)
10. Height (to indicate whether measured, reported or an alternative measure)
11. sMMSE score (for those with capacity only)

### Data collection and outcome assessments

#### Timing of assessments

Assessments were made at baseline, at 3 months and at 6 months (Table 2). All assessments were conducted within the care homes.

**Table 2:** **Assessment schedule**

Measure	Completed by	Assessment time		
		Baseline	3 months	6 months
*Nutrient intake*	*Primary researcher*	✓	✓	✓
*Height*	*Care home staff*	✓	✓	✓
*Weight*	*Care home staff*	✓	✓	✓
*BMI*	*Care home staff*	✓	✓	✓
*Handgrip strength*	*Primary researcher*	✓	✓	✓
*MAC*	*Primary researcher*	✓	✓	✓
*TSF*	*Primary researcher*	✓	✓	✓
*sMMSE*	*Care home staff*	✓	✓	✓
*VAS*	*Participant rated*	✓	✓	✓
*EQ-5D*	*Participant rated*	✓	✓	✓
*CO-OP QoL*	*Participant rated*	✓	✓	✓
*Healthcare resource usage*	*Care home staff*	✓	✓	✓
*Compliance*	*Care home staff*	✓	✓	✓

*BMI* body mass index, *MAC* mid arm circumference, *TSF* tricep skinfold thickness, *sMMSE* Standardized Mini Mental State Examination, *VAS* visual analogue scale, *EQ-5D* Euroqol 5 dimensions, *QoL* quality of life.

#### Outcome measurements

One objective of this trial is to evaluate the feasibility and acceptability of a range of quantitative outcome tools and assessment methods for use within a definitive trial (Table 3). The assessments to be evaluated included change in energy and protein intake, anthropometric parameters (weight, BMI, handgrip strength, MAMC) and patient-centric measures such as health state (EQ-5D), quality of life (CO-OP), participant-rated appetite and dietary satisfaction (VAS).

**Table 3:** **Quantitative outcome assessments**

Outcome	Completed by	Assessment
*Handgrip strength (kg)*: an index of general upper extremity strength (function)	*Primary researcher*	Measured using a handgrip dynamometer on the non-dominant arm [39].
		Limitations include the influence of debility, age and familiarity with the technique. The number of residents that refuse to participate, or for whom the measurement is not feasible, will be recorded.
*Mid arm muscle circumference (MAMC) (cm)*: an estimate of muscle mass	*Primary researcher*	Calculated using mid-upper arm circumference (MUAC) (measured with a tape measure) and tricep skinfold thickness (TSF) (measured with a standardised skinfold calliper): *MAMC (cm) = MUAC (cm) − 3.14 × TSF (cm)*
*Mean energy (kcal), protein (g) and fluid intake (ml):*	*Primary researcher*	Calculated from analysis of three non-consecutive 24-h food record charts. Usual tableware such as bowls, plates and glasses will be measured in each care home at baseline, and the size/capacity will be recorded.
		Nutrient intake will be determined using the dietary analysis software package *Diet Plan 6* (Forestfield Software Ltd, West Sussex, UK), which is installed with the complete set of UK food tables.
*Height (m)*	*Care staff measured*	Taken from clinical records or measured using a stadiometer. If standing height cannot be measured, self-reported height is considered the superior secondary method, or ulna length can be measured to obtain an estimate. Information on the method used to measure height will be collected.
*Weight (kg)*	*Care staff measured*	Measured using clinical calibrated standing, chair or hoist scales. Information on the method used to measure weight will be collected.
*Body mass index (BMI) (kg/m* ^*2*^ *)* A measure of adiposity	*Care staff measured*	Calculated using: *weight (kg)/height (m* ^*2*^ *).*
		Validity is limited by the influence of gender, ethnicity and age on body composition, which is not accounted for within the calculation [40]. Reliability is also questionable in the presence of confounding factors including oedema or ascites [41].
*Healthcare resource usage*	*Care staff measured*	The healthcare resource usage questionnaire to be piloted within this trial has been developed from consideration of existing instruments submitted for use in residential care settings on the ‘MRC Database of Instruments for Resource Use Measurement’ (DIRUM). The questionnaire will be completed by care home staff, from baseline to 3 months and from 3 to 6 months for each participating resident.
*Health state using The EuroQol-5D (EQ-5D)*	*Participant rated*	There is no malnutrition-specific measure of health state or quality of life for patients, so these well-established and validated measures will be piloted within this population to inform as to whether they are appropriate for completion by care home residents with varying cognitive function. The Euroqol group and the Dartmouth CO-OP project have granted permission to use the tools
*Quality of life using the CO-OP Quality of life chart*	*Participant rated*	
*Visual analogue scale (VAS) for self-perceived appetite and dietary satisfaction*	*Participant rated*	A VAS tool has been developed to measure the following subjective sensations; ‘hunger’, ‘appetite’, ‘dietary satisfaction’, ‘pleasantness of meals’, ‘pleasantness of snacks’ and ‘pleasantness of drinks’ and will be piloted within this trial.

### Phase 2: sequential qualitative assessment

In phase 2 of the trial, the primary researcher will employ phenomenological methodology, to gather descriptions of resident and staff experiences of phase 1 of the trial.

#### Semistructured interviews with residents

The primary researcher will use a semistructured interview approach with the residents recruited into phase 2. The interviews will be organised around a topic guide, designed to explore the perspectives of the residents on taking part in research, and the experiences of residents within the care homes randomised to all three allocated interventions. The interviews will be conducted face-to-face, to provide a means of acquiring insight and understanding of the resident experience and values [42] and are anticipated to last for 30 to 60 min for each individual.

#### Staff focus groups

The primary researcher will facilitate a focus group discussion with between two and eight staff within each participating care home, for which management approval has been received for focus group participation. This technique has been chosen as a means of obtaining information about a range of staff experiences and to highlight any variations in perspectives between the staff within each home and between care home types [43]. The groups are anticipated to last 1–2 h for the duration. It is hoped that the focus groups will generate a lot of information in a relatively short amount of time. As the staff within a care home work closely together, it is anticipated that they will be able to engage in discussion.

In light of the possibility for digression, the interviews and focus groups will be audio taped, to enable transcription for analysis. Permission to audio tape the interviews and focus groups will be obtained prior to the start of the phase 2.

#### Sample size

No formal sample size calculation was performed for this trial, as the primary outcome measures are concerned with recruitment, retention and the feasibility and acceptability of the trial [44]. Any investigations of changes in key study parameters were exploratory only. Based on the capacity of the selected care homes (29–72) and the risk of malnutrition within the UK elderly care home population (30%–42%), it was estimated that between 9 and 30 residents would be identified as at risk of malnutrition within each participating care home and would be considered for receipt of the assigned nutritional intervention. It was decided that this estimated sample size of *n* = 50 to *n* = 180 would provide sufficient data to assess trial feasibility. Effective sample size calculation for the future definitive trial will be informed by rates of recruitment and retention.

#### Cluster randomisation

Cluster randomisation took place following recruitment and baseline assessment within each care home, via the University of Birmingham Clinical Trials Unit randomisation service. Each care home was randomised using a computer-generated random number list to dietetic-led food-based intervention, dietetic-led ONS intervention or the standard care home nutritional intervention for malnutrition. Stratification by care home type ensured that one nursing home and one residential home was allocated to each intervention arm. The primary researcher communicated confirmation of the intervention allocation and the care home trial number to each participating care home.

#### Statistical methods

The Birmingham Clinical Trials Unit (BCTU) will provide support with the analysis of data. Statistical analyses will be performed using IBM SPSS, version 21.

### Phase 1 quantitative analysis

#### Descriptive statistics

As effective hypothesis testing requires a powered sample size [44], analysis will be limited to descriptive statistics and an exploratory analysis to provide estimates of key parameters and to inform the definitive trial design.

Baseline variables, as well as data on resident withdrawals, mortality, healthcare resource-usage, adverse events and compliance collected throughout the trial, will be summarised as *n* (%), mean (standard deviation) or median (interquartile range), as appropriate, to characterise the overall sample and to highlight any imbalances between the randomised trial arms. A standard CONSORT diagram (Figure 2) will be used to describe the flow of residents through the trial. This information will summarise the feasible eligibility, recruitment and 3- and 6-month follow-up rates. Potential differences in attrition rates and other data quality issues will be identified and used to inform the design of the definitive trial.

#### Statistical analyses

The detailed statistical approach will be published subsequently with the results of the trial.

All continuous data will be tested for normality using Kolmogorov-Smirnov. Categorical variables will be compared between intervention arms at baseline using the chi-square test. Mean changes in continuous outcome measures, such as nutrient intake and anthropometric parameters, will be calculated at 3 and 6 months, along with 95% confidence intervals. Group mean changes will be compared between each of the dietetic-led intervention arms and the standard care arm, using the independent-sample *t*-test for normally distributed data and the Mann-Whitney *U* test for data, which violates normality. Where sensitivity to change is suggested from the 95% confidence interval, the independent-sample *t*-test will be used to compare food-based intervention with ONS intervention for that particular outcome measure.

Completion rates and missing data will be summarised for all outcome assessments, along with estimates and variances, to determine the most appropriate primary outcome measure for a definitive trial. If an appropriate primary outcome measure is identified within this trial, the data collected will help to inform the calculation of the intracluster correlation coefficient (ICC), the design effect (DE) and the effective sample size for a definitive trial.



where *(m × k)* is the total number of subjects in a clustered trial, *m* is the number of subjects in a cluster and *k* is the total number of clusters.

### Phase 2 qualitative analysis

The qualitative data will be analysed using the Krueger [45] and Ritchie and Spencer [46] framework analyses, assisted by the NVivo computer programme as required. The process of data analysis will begin during data collection, through the effective facilitation of the interview and focus group discussions, complemented by observational notes. Following data collection, the dietitian researcher will transcribe the audio tapes and identify major themes.

Concepts, ideas and short phrases identified within the text will be used to develop categories and a thematic framework. Once a framework has been developed, the data will be indexed using a process of sorting, highlighting and arranging quotes to make comparisons between and within cases. Once indexed, the quotes will be re-arranged under the appropriate thematic content. The final stage of analysis will be mapping and interpreting the data, identifying links between the quotes and exploring and explaining patterns of association.

The qualitative analysis will be interpreted alongside the quantitative feasibility and acceptability findings, to inform the design of the future definitive trial.

## Discussion

Careful planning to overcome the challenges of UK care home-based research is essential to prepare and design a cluster randomised trial of adequate size and quality to evaluate the efficacy of nutrition support interventions. The complexities inherent in care home research has resulted in the under representation of this population in research trials. Challenges can include recruitment difficulties due to physical and/or cognitive impairments [24, 47], the consent process [24, 47] and the high attrition rates of older people from research [24]. Data collection within the busy schedule of care homes, along with poor staff compliance with the intervention and data protection protocols [24], can also pose issues for researchers. In previous nutritional intervention studies conducted within the care home setting, methodological limitations have been acknowledged, for example, difficulties with receipt of consent [14, 48], and drop-out rates of 25%–33% [27, 49, 50]. Before an adequately powered, high-quality cluster randomised controlled trial comparing nutritional interventions for malnutrition in the care home setting can be designed and conducted, a feasibility trial is being completed to provide the necessary information.

### Other practical issues involved in conducting the trial

#### Adverse events (AEs) and serious adverse events (SAEs)

This trial was considered to be low risk. The nutritional interventions are well established and are currently in use to treat malnutrition in the care home population. Expected adverse events included:

- A risk of mild side effects in response to a fortified diet or prescribed ONS, including diarrhoea, bloating, nausea and satiety.

No other risks were anticipated, and therefore it was considered reasonable to collect only targeted dietary intervention-related AEs and only serious adverse events (SAEs) requiring hospital admissions that were due to avoidable malnutrition or dehydration.

#### Reporting of adverse events and serious adverse events

The care home staff were required to inform the primary researcher if any AEs/SAEs relating to a resident’s malnutrition and/or its treatment were identified, through the completion and faxing of an adverse event form. The incidence and frequency of AEs were recorded at 3 and 6 months of intervention duration. Death from any cause was reported by care home staff on an AE form and faxed to the primary researcher.

#### Joint trial steering committee (TSC)/data monitoring committee (DMC)

The joint TSC/DMC includes the dietitian researcher, Chief Investigator of the trial and three independent members. The independent members include an independent statistician, a dietitian not involved in the trial and a member of a Patient and Public Involvement (PPI) panel care. The TSC/DMC meets every 3 months to undertake safety monitoring.

### Research governance

The conduct of the trial is in accordance with the International Conference on Harmonisation Guidance for Good Clinical Practice (ICH GCP) and the Department of Health’s Research Governance Framework (RGF).

#### Confidentiality of personal data

No personal information is being collected within this trial, with the exception of resident name and signature, for those residents that consent to take part in PROMs and individual interviews. This information is being collected on paper consent forms and is securely stored at the HEFT Nutrition Support Service office base, within locked cabinets. Residents are asked to consent to this. The trial data is entered onto a secure computer database. Only the primary researcher has access to this database. All information collected is treated as strictly confidential.

#### Long-term storage of data

In line with Good Clinical Practice guidelines, all essential documentation and data will be retained for at least 5 years.

### Strengths and limitations of this trial

A clear strength of this trial is that it aims to include care home residents with cognitive or mobility impairment, to allow for the evaluation of feasibility and acceptability with this unique and complex population, frequently at risk of malnutrition. To address the lack of good quality evidence for all reported outcomes within the care home setting, the trial proposes the evaluation of feasibility of a wide range of outcome parameters, including participant-centric outcome measures. Piloting allows for outcome assessments that are found to be infeasible or unsatisfactory to be modified in the definitive trial design. This trial provides an opportunity to develop and refine consistent practices and to improve data integrity. Refinement of documentation, recruitment and consent processes and data collection tools and methods will be informed by the outcomes of this trial, enabling for the future trial to be designed and conducted with accuracy and precision.

A limitation of this trial is that it is only being conducted within six care home sites in the Solihull area, limiting generalisability to other care home sites across the UK, where dietetic input may not be as regular or intensive. Due to the nature of the nutritional interventions under investigation, it has not been possible to blind the care home residents or staff to the allocated interventions.

## Conclusions

This protocol has defined the aims and objectives of a feasibility trial and has provided a detailed description of the interventions, the study design and the methods of data collection. For the definitive trial, the protocol will be revised to incorporate the suggestions provided by care providers. The future trial will aim to consider and compare a number of outcomes, including nutrient intake, anthropometric parameters, patient-reported outcomes and the cost effectiveness of the interventions. It is anticipated that the results of the definitive trial will inform decisions by dietitians, general practitioners, care home providers and commissioners, regarding the appropriate use of nutritional treatment for malnutrition in care homes.

## Authors’ information

Contributor RS is an MRes student at the University of Birmingham and a senior nutrition support dietitian at the Heart of England NHS Foundation trust. AR is a senior lecturer in Physiotherapy and an Academic Lead for Physiotherapy at the University of Birmingham. NI is a senior statistician and is assistant director of the Birmingham Clinical Trials Unit (BCTU). Both AR and NI are supervisors of the MRes research project. CS is a senior lecturer in Speech Therapy at University College London. CR is the Neuroscience Trials Team Leader within the Birmingham Clinical Trials Unit (BCTU).

## Electronic supplementary material

Additional file 1: **Approval letter.** Research ethics approval from the West Midlands NHS Local Research Ethics Committee. (PDF 162 KB)

Additional file 2: **Governance approval letter.** The protocol was also submitted to the Research and Development Department of the Heart of England NHS Foundation Trust for Research. (PDF 84 KB)
