# Supplementary material for: A cluster randomised feasibility trial evaluating six-month nutritional interventions in the treatment of malnutrition in care home-dwelling adults: recruitment, data collection and protocol
Source: Pilot Feasibility Stud. 2015 Jan 12;1:3. doi: 10.1186/2055-5784-1-3 (PMC5066518; doi:10.1186/2055-5784-1-3)
Supplement: Supplementary file 1 — Additional file 1: Approval letter. Research ethics approval from the West Midlands NHS Local Research Ethics Committee. (PDF 162 KB) [file 40814_2014_2_MOESM1_ESM.pdf]

NHS
Health Research Authority

NRES Committee West Midlands - Edgbaston
The Old Chapel
Royal Standard Place
Nottingham
NG1 6FS

Telephone: 0115 8839311
Facsimile: 0115 8839294

23 September 2013

Miss Ruth E Stow
Nutrition Support Service
3 The Green
Stratford Road, Shirley, Solihull
B90 4LA

Dear Miss Stow

**Study title:**	**A cluster randomised feasibility study evaluating current dietary interventions in the treatment of malnutrition in care home-dwelling adults**
**REC reference:**	**13/WM/0390**
**Protocol number:**	**N/A**
**IRAS project ID:**	**139209**

The Proportionate Review Sub-committee of the NRES Committee West Midlands - Edgbaston reviewed the above application on 18 September 2013.

We plan to publish your research summary wording for the above study on the NRES website, together with your contact details, unless you expressly withhold permission to do so. Publication will be no earlier than three months from the date of this favourable opinion letter. Should you wish to provide a substitute contact point, require further information, or wish to withhold permission to publish, please contact the Co-ordinator Miss Andrea Graham, nrescommittee.westmidlands-edgbaston@nhs.net.

Ethical opinion

- The Committee noted the study was previously given an unfavourable opinion by another Committee on the basis it should not include adults who lack capacity to consent. The full Committee indicated the study would be suitable for Proportionate Review upon resubmission
- The previous Committee asked for evidence the Care Home Management had provided consent for the study which has now been provided.
- The Committee agreed the Study meets the NMEIT criteria
- The Committee commented the randomisation is not really randomisation but a nominated treatment at each care home. However there are no concerns.

On behalf of the Committee, the sub-committee gave a favourable ethical opinion of the above research on the basis described in the application form, protocol and supporting documentation, subject to the conditions specified below.

Ethical review of research sites

The favourable opinion applies to all NHS sites taking part in the study, subject to management permission being obtained from the NHS/HSC R&D office prior to the start of the study (see "Conditions of the favourable opinion" below).

Conditions of the favourable opinion

The favourable opinion is subject to the following conditions being met prior to the start of the study.

Management permission or approval must be obtained from each host organisation prior to the start of the study at the site concerned.

Management permission ("R&D approval") should be sought from all NHS organisations involved in the study in accordance with NHS research governance arrangements.

Guidance on applying for NHS permission for research is available in the Integrated Research Application System or at http://www.rdforum.nhs.uk.

Where a NHS organisation's role in the study is limited to identifying and referring potential participants to research sites ("participant identification centre"), guidance should be sought from the R&D office on the information it requires to give permission for this activity.

For non-NHS sites, site management permission should be obtained in accordance with the procedures of the relevant host organisation.

Sponsors are not required to notify the Committee of approvals from host organisations.

It is the responsibility of the sponsor to ensure that all the conditions are complied with before the start of the study or its initiation at a particular site (as applicable).

You should notify the REC in writing once all conditions have been met (except for site approvals from host organisations) and provide copies of any revised documentation with updated version numbers. The REC will acknowledge receipt and provide a final list of the approved documentation for the study, which can be made available to host organisations to facilitate their permission for the study. Failure to provide the final versions to the REC may cause delay in obtaining permissions.

Approved documents

The documents reviewed and approved were:

Document	*Version*	*Date*
Covering Letter		11 September 2013
GP/Consultant Information Sheets	2.0	01 September 2013
Interview Schedules/Topic Guides	Staff focus group 1.0	01 July 2013
Interview Schedules/Topic Guides	Semi structured interview 1.0	01 July 2013

Investigator CV		01 September 2013
Letter from Sponsor		27 June 2013
Letter from Statistician		01 September 2013
Letter of invitation to participant	2.0	01 September 2013
Other: Unfavourable Opinion Letter		29 August 2013
Other: CV - Ruth Stow		01 September 2013
Other: CV - Natalie Ives		01 September 2013
Other: SAE	1.0	01 July 2013
Other: HPC letter		
Other: Letter regarding contract		09 January 2012
Participant Consent Form: Care Home	2.0	01 September 2013
Participant Consent Form: For questionnaires and visual analogue scales	2.0	01 September 2013
Participant Consent Form: For Interviews	1.0	01 July 2013
Participant Consent Form: Staff experience focus groups	1.0	01 July 2013
Participant Information Sheet: Care Home	2.0	01 September 2013
Participant Information Sheet: For questionnaires and visual analogue scales	2.0	01 September 2013
Participant Information Sheet: For semi structured interviews	1.0	01 July 2013
Participant Information Sheet: For focus groups	1.0	01 July 2013
Protocol	2.0	01 September 2013
Questionnaire: SMMSE		23 May 2013
Questionnaire: EQ-5D-5L	2	
Questionnaire: COOP Quality of Life Chart		
Questionnaire: VAS	1.0	01 July 2013
Questionnaire: Healthcare resource usage pilot	1.0	01 July 2013
Questionnaire: MUST	1.0	01 July 2013
Questionnaire: Care home screening log	2.0	01 September 2013
REC application	139209/498468/1/160	06 September 2013
Referees or other scientific critique report		09 July 2013
Summary/Synopsis	2.0	01 September 2013

Membership of the Proportionate Review Sub-Committee

The members of the Sub-Committee who took part in the review are listed on the attached sheet.

Statement of compliance

The Committee is constituted in accordance with the Governance Arrangements for Research Ethics Committees and complies fully with the Standard Operating Procedures for Research Ethics Committees in the UK.

After ethical review

<u>Reporting requirements</u>

The attached document "After ethical review – guidance for researchers" gives detailed guidance on reporting requirements for studies with a favourable opinion, including:

- Notifying substantial amendments
- Adding new sites and investigators
- Notification of serious breaches of the protocol
- Progress and safety reports
- Notifying the end of the study

The NRES website also provides guidance on these topics, which is updated in the light of changes in reporting requirements or procedures.

Feedback

You are invited to give your view of the service that you have received from the National Research Ethics Service and the application procedure. If you wish to make your views known please use the feedback form available on the website.
information is available at National Research Ethics Service website > After Review

**13/WM/0390**	**Please quote this number on all correspondence**

We are pleased to welcome researchers and R & D staff at our NRES committee members' training days – see details at http://www.hra.nhs.uk/hra-training/

With the Committee's best wishes for the success of this project.

Yours sincerely

Mr Paul Hamilton
Chair

Email: nrescommittee.westmidlands-edgbaston@nhs.net

Enclosures: *List of names and professions of members who took part in the review*

"After ethical review – guidance for researchers"

Copy to: *Miss Elizabeth Adey, Heart of England NHS Foundation Trust*
Dr Alison B Rushton, University of Birmingham

NRES Committee West Midlands - Edgbaston

Attendance at PRS Sub-Committee of the REC meeting on 18 September 2013

Committee Members:

Name	*Profession*	*Present*	*Notes*
Mr Paul Hamilton	Retired Local Government Officer	Yes	Chair
Dr Adrian Hamlyn	Consultant Physician & Hepatologist	Yes	
Dr Nigel Langford	Consultant Clinical Pharmacologist & General Physician	Yes	

Also in attendance:

Name	*Position (or reason for attending)*
Miss Andrea Graham	REC Coordinator
Miss Rebecca Morledge	Assistant Coordinator
