# Supplementary material for: A cluster randomised feasibility trial evaluating six-month nutritional interventions in the treatment of malnutrition in care home-dwelling adults: recruitment, data collection and protocol
Source: Pilot Feasibility Stud. 2015 Jan 12;1:3. doi: 10.1186/2055-5784-1-3 (PMC5066518; doi:10.1186/2055-5784-1-3)
Supplement: Supplementary file 2 — Additional file 2: Governance approval letter. The protocol was also submitted to the Research and Development Department of the Heart of England NHS Foundation Trust for Research. (PDF 84 KB) [file 40814_2014_2_MOESM2_ESM.pdf]

Research & Development Directorate

Medical Innovation Development Research Unit
Office Hours (Mon-Fri): 09.00 – 17.00
Tel: 0121 424 1633
Fax: 0121 424 3167
Head of Research & Innovation: Bethan Bishop
R&D Manager - Governance & Operations: Elizabeth Adey
R&D Manager - Finance & Facilities: Rachel Ward
Please send e-mails via firstname.surname@heartofengland.nhs.uk

Birmingham Heartlands Hospital
Bordesley Green East
Birmingham B9 5SS

Tel: 0121 424 2000
Fax: 0121 424 2200

17/10/2013

Miss Ruth Stow
Nutrition Support Service
3 The Green
Stratford Road, Shirley,
Solihull
B90 4LA

Dear Miss Ruth Stow

R&D Code: **2013102EM** ***Re: Study title:*** **A cluster randomised feasibility study evaluating current dietary interventions in the treatment of malnutrition in carehomedwelling adults**

EudraCT: N/A

I am pleased to inform you that the R&D review of the above project is now complete and has been formally approved to be undertaken at the following sites within Heart of England NHS Foundation Trust

Please Note: This approval only extends to Heart of England NHS Foundation Trust and not to individual care homes.

Solihull Hospital Research Site

The following documents were reviewed:

Document/Version/Date

Protocol Version 2 Dated 01 September 2013
PIS Care Home, Version 2, 01 September 2013
For questionnaires and visual analogue scales, version 2.0, 01 September 2013
For semi-structured interviews, version 1.0, 01 July 2013
For focus groups, version 1.0, 01 July 2013
ICF Care Home, version 2.0, 01 September 2013
For interviews, version 1.0, 01 July 2013 +
Staff experience focus groups, version 1.0, 01 July 2013
GP letter Version 2.0, 01 September 2013
NHS NRES Application Form Signed by Dr Alison Rushton on 01 October 2013
NRES Site Specific Information Form Signed by Miss Ruth Stow on 04 October 2013
NRES Approval Letter 23 September 2013

… continued …

MHRA notice of Acceptance (if applicable) Not applicable
Any Standard Operating Procedures for the Study
Other documents (please specify):

**Interview Schedules/Topic Guides**	Staff focus group 1.0, 01 July 2013
**Interview Schedules/Topic Guides**	Semi-structured interview 1.0, 01 July 2013

The conditions of this approval are as follows:

1) You adhere to the approved version of the protocol and notify R&D immediately of any changes to the study, including any new staff working on the project, who may require Trust or Honorary contracts issued.
2) You notify R&D immediately of any Serious Adverse Events, including Suspected Unexpected Serious Adverse Reactions (SUSARs)
3) You adhere to the requirements of the ethics committee as detailed in their approval letter and standard operating procedures which can be found on www.nres.npsa.nhs.uk
4) For CTIMP studies, you adhere to the regulations, including good clinical practice, of The Medicines for Human UK Clinical Regulations (SI 2004/1031; SI 2006/1928; SI 2006/294; SI 2008/941; SI 2009/1164; SI 2009/3063; and SI 2010/1882).
5) You notify R&D immediately of any Serious Breaches of GCP or the protocol occurring on this site. This applies to both sponsored and hosted projects. Guidance on Serious Breaches identification & reporting can be found at: http://www.mhra.gov.uk/Howweregulate/Medicines/Inspectionandstandards/GoodClinicalPractice/News/CON084915
6) You adhere to the applicable R&D Standard Operating Procedures which can be found on http://sharepoint/policies/default.aspx under R&D
7) You notify R&D on completion of the project

The duration of this approval extends to the date specified in the IRAS ethics application form, except where action is taken to suspend or terminate the opinion or should your research not begin within 2 years of the approval date.

Pharmacy

Should your study require the dispensing of drugs, please do not commence work on the project until pharmacy has issued the green light, as per MHRA requirements (http://www.mhra.gov.uk/Howweregulate/Medicines/Inspectionandstandards/GoodClinicalPractice/Frequentlyaskedquestions/index.htm). The green light confirms that pharmacy has all procedures and documentation in place and can comply with the medicines management aspects of the study. The pharmacy team will email you the green light approval once the above is in place.

May I also draw your attention to the Research Governance Framework which can be found on the internet http://www.dh.gov.uk/en/Publicationsandstatistics/Publications/PublicationsPolicyAndGuidance/DH_4108962 and remind you that all research within the Trust should be run to the standards as outlined in this document. Guidance and advice is always available from the Department of Research and Development should you require it at any stage of your project.

If you have any queries please do not hesitate to contact me.

Yours sincerely

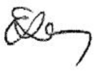

Liz Adey
Head of Research

Cc: Dr Alison B Rushton
